# Supplementary material for: Study on the Fingerprint and Atmospheric Activity of Volatile Organic Compounds from Typical Industrial Emissions
Source: Int J Environ Res Public Health. 2023 Feb 16;20(4):3517. doi: 10.3390/ijerph20043517 (PMC9965789; doi:10.3390/ijerph20043517)
Supplement: Supplementary file 1 [file ijerph-20-03517-s001.zip › ijerph-2167758-supplementary.pdf]

Supporting Information

Table S1. Summary of VOC health risk assessment

pollutants	HI				
	plastic products	Packaging and Printing	ink	furniture manufacturing	vehicle manufacturing
Pentane	5.54E-01	1.26E-01 ~ 3.72E-01	9.69E-03 ~ 1.81E-02	2.06E-02 ~ 2.35E-02	2.17E-02 ~ 2.38E-02
Hexane	4.45 E-03	1.63E-02 ~ 1.20E-01	2.75E-04 ~ 7.34E-03	/	/
Heptane	7.14 E-03	2.93E-02 ~ 1.22E-01	1.45E-02 ~ 4.54E-01	2.07E-02 ~ 2.90E-02	1.30E-03 ~ 3.29E-03
Nonane	3.99 E-02	4.97E-02 ~ 6.77E-02	3.24E-02 ~ 6.11E-02	0.00E+00 ~ 1.14E-02	/
1,3-Butadiene	5.78 E-02	3.48E-02 ~ 4.91E-01	5.20E-04 ~ 1.68E-02	0.00E+00 ~ 5.55E-03	/
methyl chloride	1.13 E-02	5.36E-03 ~ 7.33E-03	2.36E-03 ~ 5.61E-03	1.22E-04 ~ 3.96E-04	/
1,1-Dichloroethylene	9.96E-05	4.25E-05 ~ 6.41E-04	2.17E-05 ~ 7.95E-05	/	/
carbon tetrachloride	1.53E-03	1.38E-03 ~ 1.61E-03	2.62E-03 ~ 1.04E-01	2.56E-04 ~ 4.44E-04	6.76E-05 ~ 4.44E-04
1,2-Dichloropropane	2.10E-01	9.57E-02 ~ 1.53E-01	2.05E-01 ~ 4.02E-01	0.00E+00 ~ 3.27E-03	/
1,1,2-Trichloroethane	2.93E-01	1.27E-01 ~ 6.14E-01	3.26E-01 ~ 6.01E-01	/	/
Tetrachloroethylene	6.76E-02	2.60E-02 ~ 5.12E-02	4.18E-03 ~ 1.06E-02	5.06E-03 ~ 1.06E-02	/
chlorobenzene	2.53E-02	3.28E-03 ~ 6.33E-03	1.39E-03 ~ 3.71E-03	/	/

m-dichlorobenzene	3.38E-04	3.95E-04 ~ 3.78E-03	/	/	/
p-dichlorobenzene	1.71E-04	1.07E-04 ~ 2.86E-04	0.00E+00 ~ 2.73E-04	/	/
o-Dichlorobenzene	8.32E-05	2.54E-05 ~ 1.03E-03	0.00E+00 ~ 2.47E-04	/	/
benzene	4.74E-01	1.85E-01 ~ 6.76E-01	4.67E-02 ~ 2.78E-01	5.08E-03 ~ 1.54E-02	4.20E-03 ~ 4.79E-03
Toluene	1.56E-02	2.48E-02 ~ 6.43E-02	1.97E-02 ~ 5.41E-01	5.00E-03 ~ 6.38E-03	/
Ethylbenzene	2.37E-02	2.21E-02 ~ 3.72E-02	4.68E-03 ~ 2.10E-02	6.54E-04 ~ 4.40E-03	/
m-paraxylene	3.21E-01	3.52E-01 ~ 5.73E-01	3.28E-02 ~ 2.45E-01	0.00E+00 ~ 3.62E-02	/
Styrene	7.52E-02	2.82E-03 ~ 6.06E-03	8.09E-03 ~ 2.18E-02	0.00E+00 ~ 2.10E-03	/
O-xylene	2.62E-01	2.14E-01 ~ 3.63E-01	8.60E-03 ~ 1.94E-01	0.00E+00 ~ 4.86E-03	/
